# Device for automated aseptic sampling: Automated sampling solution for future cell and gene manufacturing

**DOI:** 10.3389/fbioe.2024.1452674

**Published:** 2024-11-20

**Authors:** Liu Dan, Wu Ying Ying, Akshaya V. Prabhu, Ahmad Amirul bin Abdul Rahim, Zach Lee Jia Sheng

**Affiliations:** Bioprocessing Technology Institute (BTI), Agency for Science, Technology and Research (A*STAR), Singapore, Singapore

**Keywords:** cell and gene therapy, cell manufacturing, automated sampling, aseptic sampling, process analytical technology, automation

## Abstract

Cell sampling is a key step performed regularly throughout the cell manufacturing process to gather cell samples for cell growth, progress, and characteristics analysis. While the current method of sampling by pipetting in a biosafety cabinet is commonly used, it is labour-intensive and susceptible to contamination risks. We have developed Device for Automated Aseptic Sampling (DAAS), to enable automated, small volume (0.02–1.00 mL) aseptic sampling with minimal dead volume primarily for cell and gene therapy manufacturing. The aim of DAAS is to enable an accurate and consistent sampling process, with minimal contamination risks and interruption to the cells in culture. DAAS can potentially interface with other automated solutions to enable automated and streamlined cell manufacturing workflow and reduce overall manufacturing costs. DAAS has been verified as an aseptic sampling solution via repeated microbial ingression tests. It has also been tested for achieving comparable cell density and viability compared to manual pipetting, with negligible cross-sample carryover when used to sample Jurkat cells of different cell concentrations. The application of using DAAS to sample cell periodically and monitor cell growth and viability continuously for prolonged cell culture was successfully demonstrated with Jurkat cell culture in a static culture flask and donor T cell culture in an automated bioreactor system over a culture duration of 10 days in a Biosafety Level-2 laboratory. Overall, DAAS presents great potential as an automated and aseptic sampling solution, offering cell and gene therapy manufacturers easier and more frequent access to cell samples with minimal interruptions to the cell culture. This enables close monitoring of cell culture and a more automated, connected and cost-effect cell and gene therapy manufacturing process.

## Introduction

Autologous cell and gene therapy has been demonstrated to be a highly efficacious therapeutic modality with clear clinical advantages over traditional treatments for cancers and other diseases. Its efficacy is evident from the past clinical trials, and seven Chimeric Antigen Receptor T (CAR-T) and T-cell receptor T (TCR-T) cell therapy products that have been approved by FDA for use in treatment (Schuster et al., 2019; Si Lim et al., 2021; Neelapu et al., 2017; Abramson et al., 2020; U.S. Food and Drug Administration, 2024b).

At present, the demand for autologous cell and gene therapy products for medical treatment and clinical trials far outweighs the manufacturing capacity, leading to long waiting times as well as very high treatment costs (hundreds of thousands of dollars) for a single infusion for patients (Palani et al., 2023; Elsallab and Maus, 2023). This can be largely attributed to the inefficient manufacturing process, which involves multiple variables from starting patient cell source, cell culture condition, to final cell population, with each group of manufactured cells as unique as the patients (Ayala et al., 2024). It is widely accepted that one of the ways forward to improve manufacturing capacity and significantly reduce treatment costs is to adopt standardized and automated manufacturing (Abou-El-Enein et al., 2021; Blache et al., 2022).

Meanwhile, key manufacturing guidelines and multiple product release criteria including identity, purity, potency, and sterility from the regulatory bodies must be fulfilled for manufacturers to qualify the end product prior to infusion into the patient (U.S. Food and Drug Administration, 2024a). Regular cell monitoring to obtain critical cell information such as cell phenotype and potency during the manufacturing are key to navigating the complexities of the unpredictable manufacturing process, enabling adaptive in-process control and manufacturing cell and gene therapy products that can fulfill the release criteria (Mgebroff, 2021). State-of-art automated cell manufacturing systems like the CliniMACS Prodigy^®^ (Miltenyi Biotec, Bergisch Gladbach, Germany) and Cocoon^®^ Platform (Lonza, Basel, Switzerland) require users to extract cell samples for offline cell analysis (Miltenyi Bioindustry, 2024; Lonza, 2024).

Altogether, the implementation of automated cell monitoring represents an important catalyst for future cell and gene therapy manufacturing. One essential enabler for this implementation is automated cell sampling technology, which however has not been adequately addressed today.

Commonly, cell sampling is performed by pipetting in a biosafety cabinet (BSC) if cells are cultured in open culture vessels, or syringes through sampling tubing if cells are cultured in closed bioreactor systems. Such manual processes are labour-intensive and susceptible to contamination risks. On the other hand, automated aseptic sampling systems that are currently in the market include NUMERA^®^ (SECURECELL^©^, AG, Switzerland), Seg-Flow S3 (Flownamics^®^, Madison, Wisconsin, United States) and MAST^®^ (Merck™, Darmstadt, Germany), bioPROBE (bbi-biotech, Berlin, Germany), and BioProfile^®^ FLEX2 On-Line Autosampler (Nova Biomedical, Waltham, United States). While they have been validated to support sterile and automated sampling, they do not fully meet the requirements for autologous cell and gene therapy manufacturing. For example, they typically require a minimum sampling volume of 1–2 mL, which could lead to considerable product loss for frequent sampling. In addition, a few of them have large footprints and require considerable physical space to operate, making their integration into cell and gene therapy manufacturing workflow and environment challenging.

**FIGURE 1:**
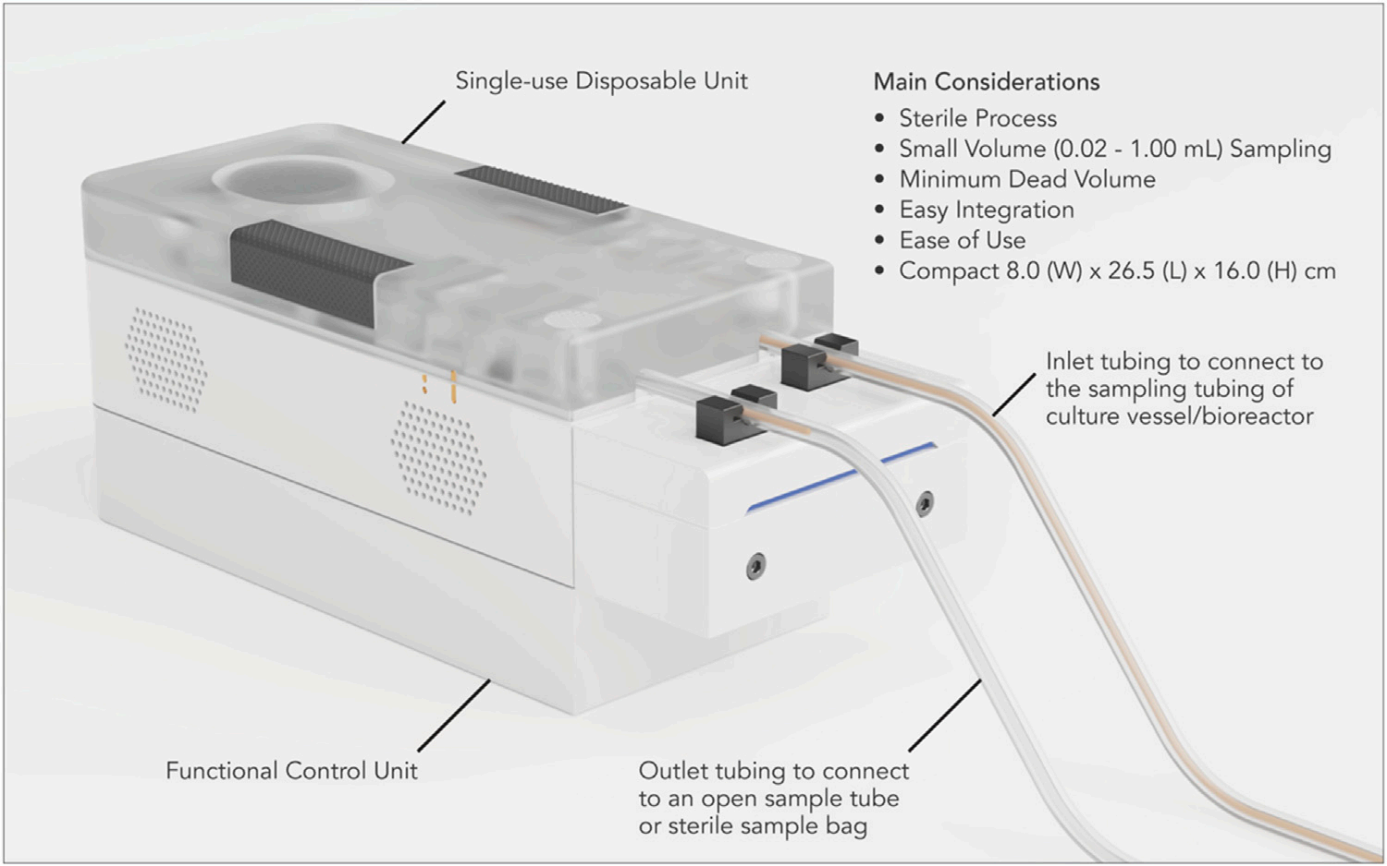
Device for automated aseptic sampling (DAAS), a compact and automated aseptic sampling device for cell and gene therapy manufacturing. It can automatically, regularly, and aseptically extract sample volumes of 0.02–1 mL from culture vessels and bioreactors into open collection tubes and sterile sample bags, with minimum dead volume and disruption to the cell culture.

In this manuscript, we describe our work in developing and verifying Device for Automated Aseptic Sampling (DAAS) (Figure 1), an automated sampling solution for autologous cell and gene manufacturing. DAAS can automatically, regularly, and aseptically extract sample volumes of 0.02–1.00 mL from culture vessels and bioreactors into either open sample tubes or closed sample bags, with minimum dead volume and disruption to the cell culture.

## Materials and methods

### Use of DAAS

We have developed a functional prototype system for DAAS, which consists of a single-use disposable unit, a functional control unit, and a Graphical User Interface (GUI) (Figure 1). The single-use disposable unit dictates the fluid flow path for culture samples. It can be sterilized through autoclaving or gamma irradiation. The functional control unit executes the sampling function to drive sample movement in the single-use disposable unit with pump and pinch valves. The sampling function was programmed with an Arduino^®^ ATmega2560 microcontroller using the Arduino^®^ (Arduino^®^, Turin, Italy) Integrated Development Environment (IDE). The GUI was developed using Python™ IDE (Python™, Delaware, United States).

The single-use disposable unit is designed to support a complete cell culture run, accommodating multiple sampling events throughout the process.

Prior to the cell culture run, the inlet tubing of the single-use disposable unit was connected to the sampling tubing of the culture vessel/bioreactor via Luer lock connection in a Class II BSC. The outlet tubing of the disposable unit was connected to an open centrifuge tube, unless otherwise specified (Figure 1). The tubing connection between DAAS and the culture vessel/bioreactor was maintained through the cell culture run.

Automated cell sampling was carried out via DAAS at specified time points of the cell culture run and samples were collected into the centrifuge tube attached to the outlet tubing of the disposable unit. After each sampling, the centrifuge tube was replaced with a new one. At the end of the cell culture run, the single-use disposable unit was disconnected from the culture vessel/bioreactor and disposed of.

### Bacterial ingression testing

This experiment was performed inside a Class II BSC.

Sterile stock tryptic soy broth (TSB), CM0129 (ThermoFisher Scientific, United States) was prepared in a 500 mL glass bottle. The bottle cap had three tubes: the first extended to the bottom of the bottle at one end and had a swabbable valve on the other end, the second extended to the bottom of the bottle at one end and was connected to DAAS via swabbable value at the other end, the third extended only halfway down the bottle without touching the TSB and was fitted with a SuperPure™ sterile hydrophobic PTFE gas filter with 0.22 µm pore size (Membrane Solutions, LLC, Shanghai, China) at the other end.

**FIGURE 2:**
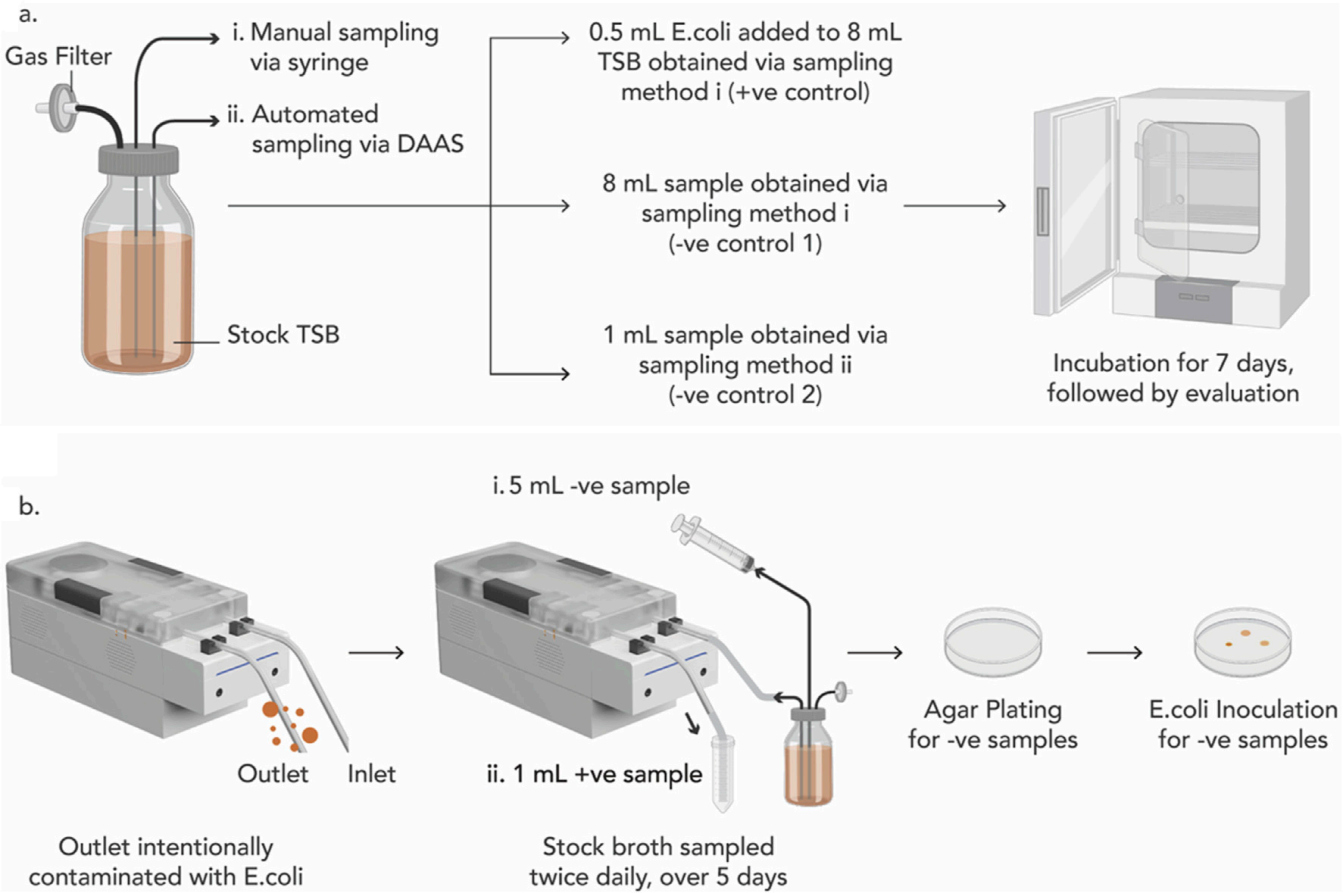
**(A)** Experiment schematics to qualify the viability of the Sterile tryptic soy broth (TSB) for *E. coli* growth (+ve control), and the sterility of the TSB (−ve control 1) and the single-use disposable unit of the DAAS (−ve control 2). **(B)** Experiment schematics for Bacterial Ingression Testing to verify DAAS’s capability to perform aseptic sampling.

Tests were first performed to qualify the stock TSB and the single-use disposable unit of DAAS (Figure 2A). 8 mL of sterile TSB was manually extracted from the stock glass bottle via the first tubing with a syringe and added with 0.5 mL of *Escherichia coli* BL21 (DE3) competent cells, C2527H (New England Biolabs GmbH, Frankfurt, Germany) suspension (∼11.5 × 10^8^ cfu/mL) as the positive control (+ve control). Another 8 mL sterile TSB was manually extracted from the stock bottle via the first tubing with a syringe as the 1^st^ negative control (−ve control 1). An additional 1 mL of sterile TSB was extracted from the stock glass bottle via the second tubing with DAAS as the 2^nd^ negative control (−ve control 2). All the extracted samples were then incubated at 37°C for 7 days. At the end of 7 days, presence of bacterial growth in the positive control would qualify the TSB for viable bacterial growth, and absence of bacterial growth in the negative controls would qualify the TSB and the single-use disposable unit of DAAS as sterile.

Upon the qualification of the stock TSB and the single-use disposable unit of DAAS, the bacterial ingression testing was performed.

One day prior to the bacterial ingression testing, the outlet tubing of DAAS was infused with 0.5 mL of *E. coli* suspension (∼11.5 × 10^8^ cfu/mL) (Figure 2B). Starting from Day 1, 5 mL of TSB was manually extracted from the stock TSB bottle via the first tubing with a syringe as B# sample (−ve sample), and 1 mL of TSB was then automatically sampled via the second tubing with DAAS as S# sample (+ve sample). This sampling was repeated twice each day, once in the morning and again in the evening for a total of 5 days, to mimic the multiple sampling events in a cell culture run. All the samples were incubated in the incubator at 37°C for 7 days. At the end of 7 days, the presence of *E. coli* in S# samples would indicate continuous contamination of the outlet tubing of DAAS with *E. coli* and the viability of the TSB. The absence of *E. coli* in B# samples would indicate that the DAAS was able to maintain the sterility of the stock TSB.

Agar plating was performed to further verify the sterility of the stock TSB after DAAS sampling. 0.5 mL of each B# sample was agar plated and incubated at 37°C for 7 days. At the end of 7 days, the absence of *E. coli* in the agar plates would indicate the sterility of the stock TSB. 0.5 mL of *E. coli* was then inoculated into each agar plate and incubated at 37°C for another 7 days. At the end of the 7 days, the presence of *E. coli* in the agar plates would indicate the viability of the agar plate for E. coli growth. Contaminated S# samples, and sterile stock TSB would indicate that the DAAS was successful in performing aseptic sampling when its outlet tubing was exposed to contamination.

### Sampling from cell solutions of different cell concentrations

Jurkat cells, Clone E6-1 (ATCC, VA, United States) at a density of 0.25 × 10^6^ cells/mL, in 50 mL complete RPMI 1640 media, A1049101 (ThermoFisher, MA, United States) with 10% foetal bovine serum (FBS) (Hyclone, UT, United States), were cultured in a 75 cm^2^ U-shaped canted neck T75 cell culture flask with vented cap (430641U (Corning, NY, United States)) at 37°C, 5% CO_2_, >99.0% humidity inside the incubator for 7 days. At the end of 7 days, the entire culture volume of the flask was transferred into a 50 mL tube, and centrifuged at 130 g, for 5 min. The spent media was thereafter removed from the tube and the cells were diluted with 10 mL fresh media. A cell count was performed using an EVE automated cell counter (NanoEntek, Seoul, Korea), and the cells were further diluted into 5 target study concentrations of 10, 7, 5, 1, 0.25 × 10^6^ cells/mL accordingly, with each having 5 mL volume, in a 50 mL tube.

Automated sampling of 0.10 mL sample from the tube with cell solution of 10 × 10^6^ cells/mL concentration was performed with DAAS. This was followed by manual sampling of 0.10 mL sample using a Research Plus 10–100 µl pipette (Eppendorf, Hamburg, Germany). These two steps were repeated 3 times and followed sequentially by sampling with cell solutions of other decreasing concentrations. Extracted samples were stained with Trypan Blue, 0.4% (ThermoFisher, MA, United States) in 1:1 ratio, and the live cell concentration and viability were recorded using the EVE automated cell counter. Samples were diluted before staining with Trypan Blue if they were beyond the optimal testing range of the counter (1 × 10^5^–4 × 10^6^ cells/mL). All the sampling were performed in a Class II BSC.

Statistical analyses were performed using Microsoft Excel (Office 365). Paired Student’s two-tailed t-test was performed to assess the statistical significance of differences between automated and manual sampling groups. A p-value of <0.05 indicates a significant difference.

### Sampling during prolonged cell culture

#### Study 1: Jurkat cell expansion in an open culture vessel over 10 days

On Day 0, 2 sets of 50 mL Jurkat cell solutions were prepared at 0.25 × 10^6^ cells/mL in fresh complete media (RPMI 1640 media, A1049101 (ThermoFisher, MA, United States) with 10% FBS (Hyclone, UT, United States)). Each set was seeded into a sterile polypropylene jar (#310123, SPL Life Sciences, Pochon, South Korea) with lid equipped with a sterile gas filter. The first jar was further modified with an additional tubing of ID Ø1.588 mm which extended to the bottom of the jar at one end and was connected to DAAS at the other end. Both jars were placed inside the CO_2_ incubator, at 37°C, 5% CO_2_, >95% humidity, for static culture of 10 days. DAAS was located outside of the incubator on a workbench while being connected to the first jar with tubing.

**FIGURE 3:**
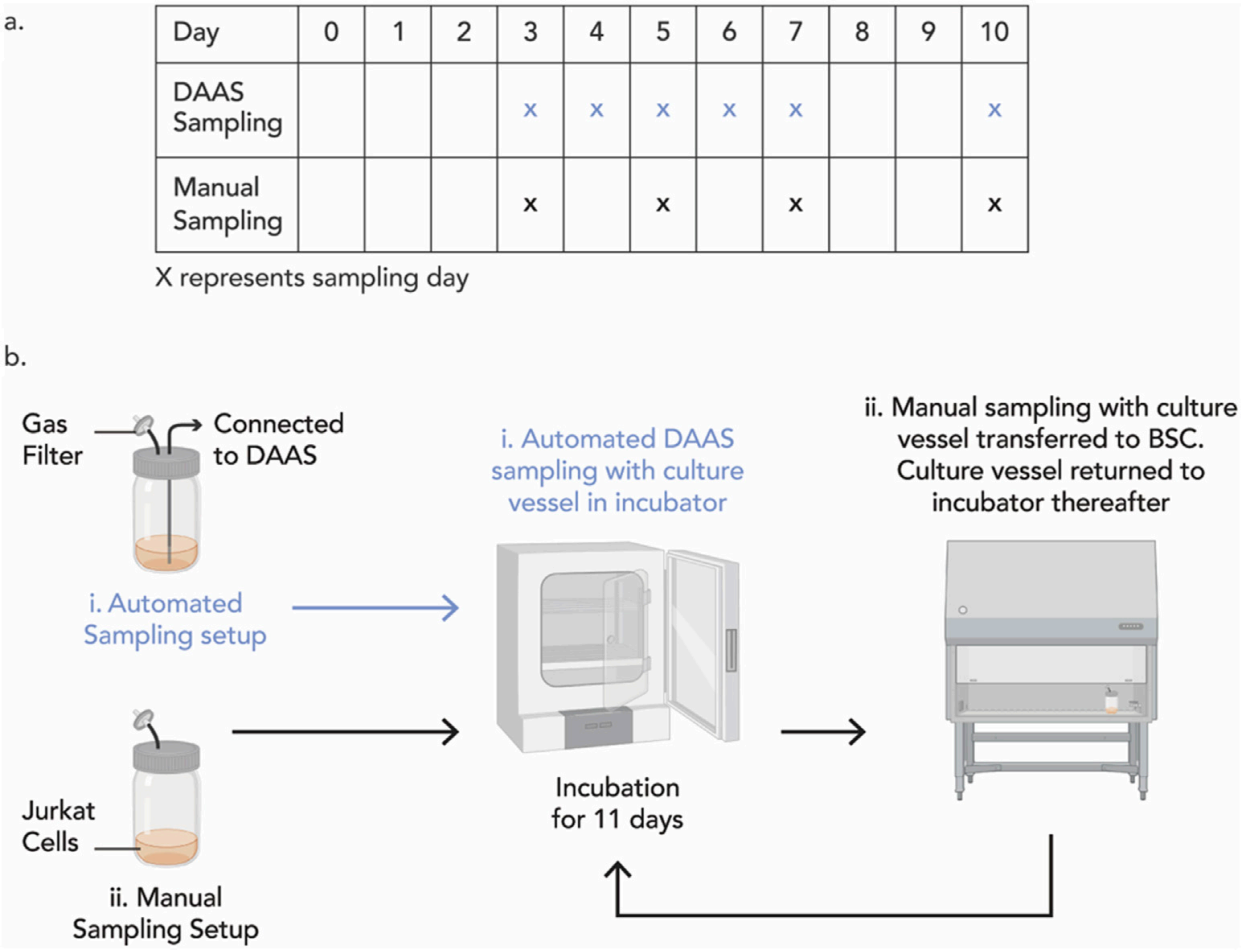
**(A)** Experiment schedule of DAAS sampling (using DAAS with culture vessel remaining in incubator) and manual sampling (using pipette with culture vessel transferred from incubator into BSC) for Study 1. **(B)** Schematic illustrating the different workflows between DAAS sampling and manual sampling for Study 1.

Daily automated sampling of 0.10 mL to the first jar was performed with the DAAS, starting from Day 3. The jar was rocked gently for 10 times while maintaining in the incubator, after which the incubator door was closed, and the sample extracted using DAAS into an open microcentrifuge tube. Manual sampling of 0.10 mL to the second jar was performed every 2–3 days, starting from Day 3. The jar was brought into the BSC, rocked gently for 10 times and 0.10 mL sample was then pipetted out (Figures 3A,B). Extracted samples were stained with Trypan Blue, in 1:1 ratio and the live cell concentration and viability were recorded using the EVE automated cell counter. Samples were diluted before staining if they were beyond the optimal testing range of the counter.

On Day 5, a complete media change was performed. Cell solutions were collected and spun down at 130 g for 5 min. Cell pellets were resuspended in 50 mL of fresh complete culture medium and seeded back into the respective jars.

#### Study 2: Peripheral blood mononuclear cells (PBMCs) derived T cell expansion in an automated and closed bioreactor system over 10 days

**FIGURE 4:**
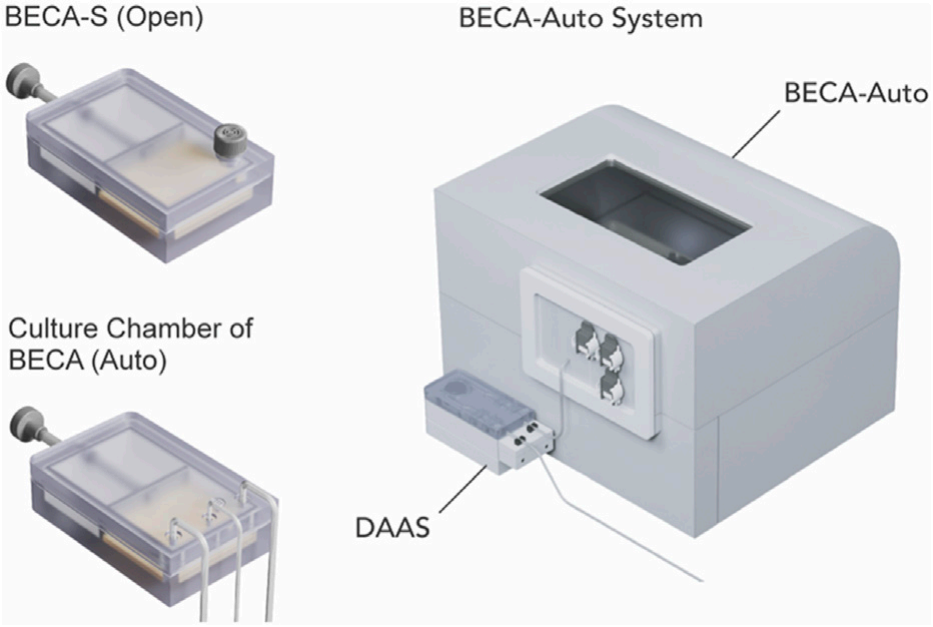
Donor T cells were cultured in Bioreactor with Expandable Culture Area Automated (BECA-Auto) (right), an automated and closed bioreactor system, and Bioreactor with Expandable Culture Area-Single-Open (BECA-S (Open)) (left top), an open culture vessel that mimics the culture chamber of BECA-Auto (left bottom). Culture in BECA-Auto was sampled through DAAS (right), and culture in BECA-S (Open) was sampled manually via pipetting in BSC.

PBMCs (STEMCELL Technologies, Canada) was prepared in complete culture medium (RPMI-1640 (ThermoFisher Scientific, MA, United States) with 10% heat inactivated FBS (HyClone), 1x GlutaMAX (ThermoFisher Scientific, MA, United States)), with supplementation of 50 IU/mL Interlukin-2 (IL-2) (STEMCELL Technologies, Canada)) at 1 × 10^6^ cells/mL. Bioreactor with Expandable Culture Area Automated (BECA-Auto), an in-house developed automated and closed bioreactor system, was used for the cell culture (Figure 4). The inlet tubing of DAAS was connected to the sampling tubing of BECA-Auto via Luer lock connection in a Class II BSC before cell culture started. All the fluid handling steps for BECA-Auto were automated and culture environment self-maintained within the equipment at 37°C, 5% CO_2_ and >90% humidity. Bioreactor with Expandable Culture Area-Single-Open (BECA-S (Open)), an open culture vessel mimicking the culture chamber of BECA-Auto was used to culture the same donor cells (Figure 4). Two different donor cells were used for two separate runs.

On Day 0, 9.5 × 10^6^ PBMCs were loaded into the culture chamber of BECA-Auto and BECA-S (Open) separately and cultured in static for 10 days 25 μL/mL ImmunoCult™ Human CD3/CD28 T Cell Activator (STEMCELL Technologies, Canada) was added on Day 0 for cell activation. Cells were automatically sampled from BECA-Auto via DAAS, and manually sampled from BECA-S via pipetting on Days 3, 5, 7, and 10. The culture area of the culture chamber was expanded on these days to bring down the cell density to 1 × 10^6^ cells/cm^2^ whenever the cell density was found to exceed 1 × 10^6^ cells/cm^2^. Complete media supplemented with 50 IU/mL IL-2 was added on the sampling day to maintain the media height at 5 mm (Day 3), or 10 mm on Days 5 and 7.

Extracted samples were stained with Trypan Blue, in 1:1 ratio and the live cell concentration and viability were recorded using hemocytometer. Samples were diluted before staining if they were beyond the optimal testing range of the hemocytometer.

## Results and discussion

### Development of DAAS

DAAS has been developed as an automated sampling solution primarily for autologous cell and gene therapy manufacturing. It addresses the important considerations for this application, namely process sterility, small volume sampling, minimal sample waste, easy integration, ease of use, and compactness through several key design features.

**FIGURE 5:**
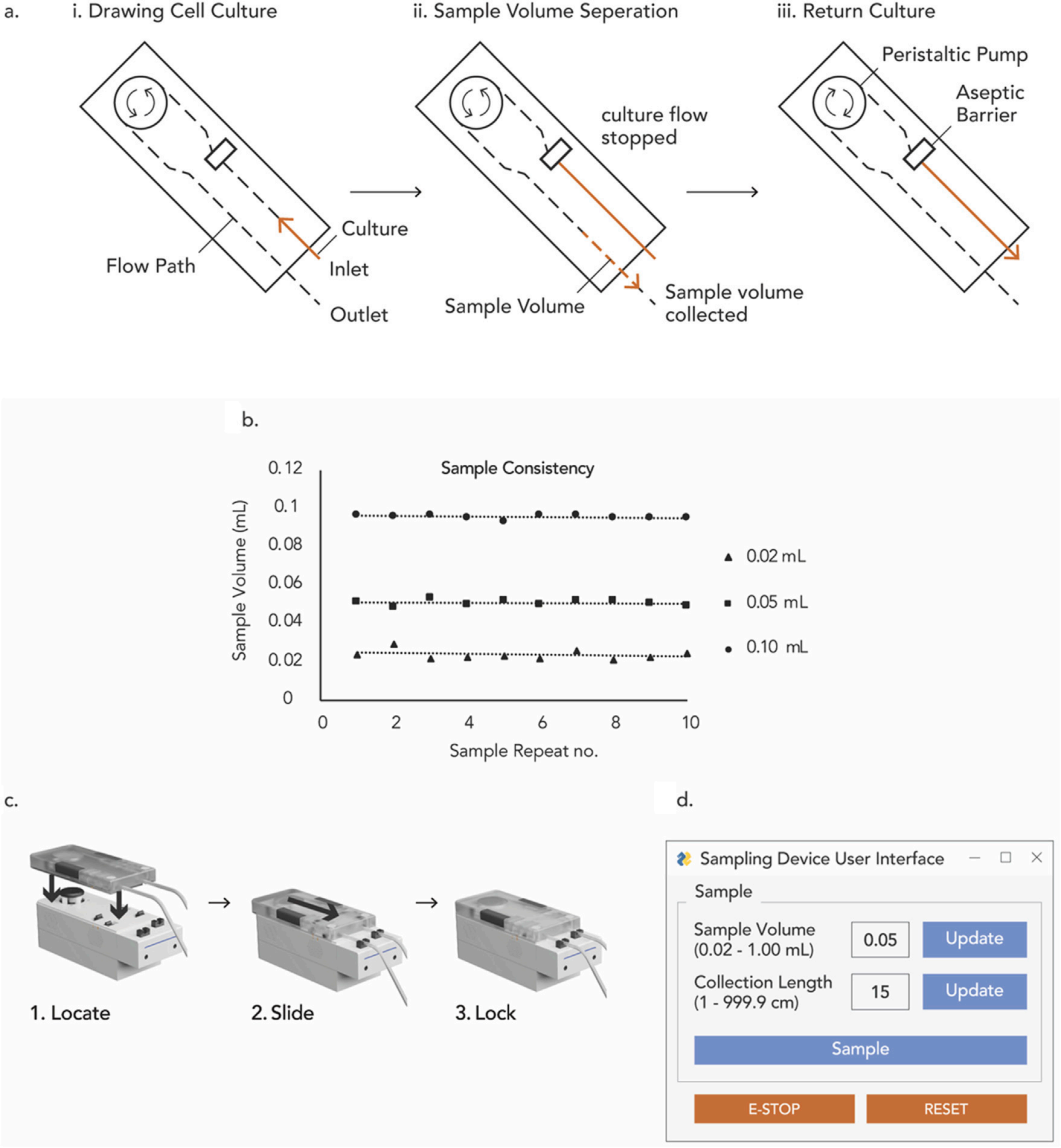
**(A)** Main flow sequence of DAAS, **(B)** volume consistency for repeated small volume sampling with DAAS, **(C)** 3-step assembly of DAAS, **(D)** GUI of DAAS.

Process sterility is paramount for cell and gene therapy manufacturing as nonsterile processes could cause contamination to the culture leading to irremediable production failure. To achieve process sterility, DAAS employs a single-use disposable unit and confines the sample flow to the tubing manifold of the disposable unit throughout the sampling process. The cell culture is thus never exposed to potential contaminations of the external environment. DAAS also establishes and upholds an aseptic barrier within the fluid path of the disposable unit by regulating the opening and closing of several pinch valves. The fluid path from the aseptic barrier to the connected culture vessel remains constantly sterile (Figure 5A). These two features enable DAAS to maintain the sterility of the connected culture vessel irrespective of the sterility condition of DAAS’ outlet tubing.

For autologous cell and gene therapy manufacturing where the total culture volume can be small, small volume sampling can be essential to enable regular cell sampling without causing significant loss of the precious culture. DAAS supports small volume (0.02–1.00 mL) sampling through optimized configuration of its single-use disposable unit, use of pump component of high resolution, and the strategical allocation and control of the regulating pinch valves. Compared to commercial automated sampling systems which typically require a minimum sampling volume of 1–2 mL, DAAS is advantageous in providing users the options for sampling below 1 mL and minimizing sample waste.

To facilitate accurate volume sampling, DAAS’ single-use disposable unit features two tubing sizes: ID Ø3.175 mm for sampling volumes of 0.20–1.00 mL, and ID Ø1.588 mm for sampling volumes of 0.02–0.20 mL. In-house test with the DAAS prototype indicated a maximum deviation of 2.5 μL at the lowest sample volume of 0.02 mL (Figure 5B). This deviation diminished as the sample volume increased, with 1.4 µL and 1.2 μL at 0.05 mL and 0.10 mL sample volumes respectively (Figure 5B). The sampling volume accuracy and consistency of DAAS have been observed to vary with the relative position of DAAS to the culture vessel/bioreactor, sampling rate and volume, and length of the connection tubing with culture vessel/bioreactor. Hence a volume calibration to optimize the DAAS operation for specific experiments is necessary.

To extract only the intended sample volume without culture loss, DAAS adopts a special flow sequence to aseptically return the excess culture to the culture vessel or bioreactor. Specifically, DAAS halts the culture flow once a desired culture volume surpasses the aseptic barrier in the tubing manifold of the single-use disposable unit. It then separates the desired culture volume from the culture flow at the aseptic barrier through the introduction of sterile air and moves the separated culture volume towards the outlet tubing. After dispensing the sample volume, DAAS returns the remaining culture in the tubing preceding the aseptic barrier to the culture vessel using sterile air (Figure 5A). Due to the presence of aseptic barrier, the culture preceding the aseptic barrier is sterile and returning it to the culture vessel would not introduce contamination. This culture return feature allows DAAS to sample with almost zero dead volume. Setting the pump speed to a low level to reduce turbulence in liquid flow and prevent inadvertent flow interruption has also shown to be useful for minimizing dead volume.

Current cell and gene therapy manufacturing processes utilize a variety of culture vessels and bioreactors. New manufacturing technologies should facilitate easy and flexible integration with these existing manufacturing platforms. The inlet tubing of DAAS can be fitted with a Luer lock to connect with culture vessels whose sampling tubings are pre-fitted with Luer locks (e.g., G-REX^®^ closed system, Xuri™ Cellbag™) via Luer lock connection. The Luer lock connection of tubing must be performed in a sterile environment such as a Class II BSC to ensure process sterility. DAAS can also be configured with weldable inlet tubing to enable tube welding for tubing connection with culture vessels. The outlet tubing of DAAS can be connected to an open sampling tube when upholding the sample sterility is not required, such as for cell count or immunostaining. Alternatively, it can be configured with a Luer lock or weldable tubing to connect to closed sample bags when sample sterility must be maintained, such as for sterility test or for subsequent sample culture.

Systems that are easy to use could help reduce human error and improve the operation efficiency. To facilitate ease of use, the assembly of DAAS was designed with a straightforward 3-step procedure (Figure 5C). DAAS features a minimalistic GUI that requires just three inputs for process specification: the length of the collection tubing, the sample volume, and the “start” trigger to initiate the sampling event. (Figure 5D). Additionally, DAAS provides a basic safety function that prioritizes culture safety in the event of a system error. If an error occurs, DAAS freezes the sampling process and closes all pinch valves to preserve the integrity of the culture. It also provides E-STOP and RESET functions for troubleshooting, allowing operators to safely return any remaining sterile culture volumes in the fluidic path to the culture vessel and proceed to the next course of action.

The clean room space for cell and gene therapy manufacturing is highly expensive. Small footprint of operation equipment helps save the facility cost and facilitates system integration into the existing manufacturing workflow. DAAS has a physical size of 8.0 cm Width × 26.5 cm Length × 16.0 cm Height. Compared to most commercial automated cell sampling systems, DAAS is notably more compact, making it highly portable for site sampling and an ideal choice for the space-constricted cell and gene therapy manufacturing environment.

### Bacterial ingression testing

Sterile sampling is the primary requirement for DAAS. This study was performed to verify the ability of the DAAS to sample aseptically and maintain the sterility of the culture vessel when its outlet tubing was exposed. It simulates the applications when sterility is not required for sample collection, for example when cells are collected for cell counting and immunostaining. For such applications, DAAS’ outlet tubing is connected to an open collection tube or well.

In the qualification test, positive control (+ve control) presented bacteria growth, indicating TSB was viable for contamination with *E. coli.* Both negative controls (−ve control 1 and 2) were absent of bacteria growth, indicating both the stock TSB and the singe-use disposable unit of DAAS were sterile (−ve control 2). These controls were further plated on agar plates for more robust contamination check, and the positive control remained positive, while the two negative controls remained negative. These results indicated that the TSB and the single-use disposable unit of DAAS were suitable for the subsequent bacterial ingression testing.

In the bacterial ingression testing, all B# samples (−ve samples) stayed clear indicating the absence of *E. coli* growth. All S# samples (+ve samples) turned turbid, indicating *E. coli* growth. The B# samples were then plated on agar plates to verify the absence of *E. coli* contamination; this was confirmed by the absence of any bacterial colonies after 7 days’ incubation. The B# agar plate samples were then inoculated with 0.5 mL *E. coli* suspensions to ascertain the viability of the agar plate for bacterial growth, and this was confirmed by the presence of bacterial colonies after 7 days of incubation. These results indicate that DAAS was able to maintain the sterility of the stock TSB while its sample outlet was purposely contaminated with *E. coli.*

To confirm the repeatability of the result, another bacterial ingression testing was performed with DAAS located outside of the BSC in a BSL 2 lab. The outlet tubing of DAAS was connected to a stock broth in a shaking incubator. The outlet tubing was branched into three extended outlets with one connected to a sterile collection tube and two connected to open centrifuge tubes. Sampling was performed through DAAS twice a week for 3 weeks (6x total sampling events). One day before each sampling day, the extended outlets that were connected to open centrifuge tubes were inoculated with *E. coli* mixture. For each sampling, a first sample volume was extracted from the stock broth into the sterile collection tube, followed by another two sample volumes extracted and collected into the open centrifuge tubes. The collected samples were then incubated and checked for bacteria growth based on observation of the broth and agar plate. The results showed that all the 6 samples collected into the sterile collection tubes were absent of, and the 12 samples collected into the open centrifuge tubes were positive for bacteria growth. These results indicated the stock broth samples remained sterile throughout the period of 3 weeks with repeated sampling with DAAS whose outlet tubing was purposely exposed.

The repeated bacterial ingression test proved DAAS was robust in performing aseptic sampling without compromising the sterility of the culture vessel even when its tubing outlet was exposed to heavy contamination. Notably, DAAS is intended to be used in a Grade B or C clean room environment; the possible viral load exposed to its tubing outlet during its application is expected to be much lower than that in the bacteria ingression tests. Additionally, when the tubing outlet of DAAS is connected to a closed sample tube or vial instead of an open centrifuge tube, the entire sampling process occurs in a fully closed environment, providing an even higher level of assurance for process sterility.

### Sampling from cell solutions of different cell concentrations

This study was conducted to evaluate DAAS for sampling accuracy in terms of cell density and viability. As sampling performance could possibly vary with cell concentrations, 5 different cell concentrations were used and sampling was repeated 3 times for each concentration. The cell concentration range 0.25–10 × 10^6^ cells/mL was chosen to cove the common cell concentration range for T cell culture. The study also involved repeated sampling without flushing of the tubing manifold pre-/post-sampling to study potential sample carryover between sampling events.

**FIGURE 6:**
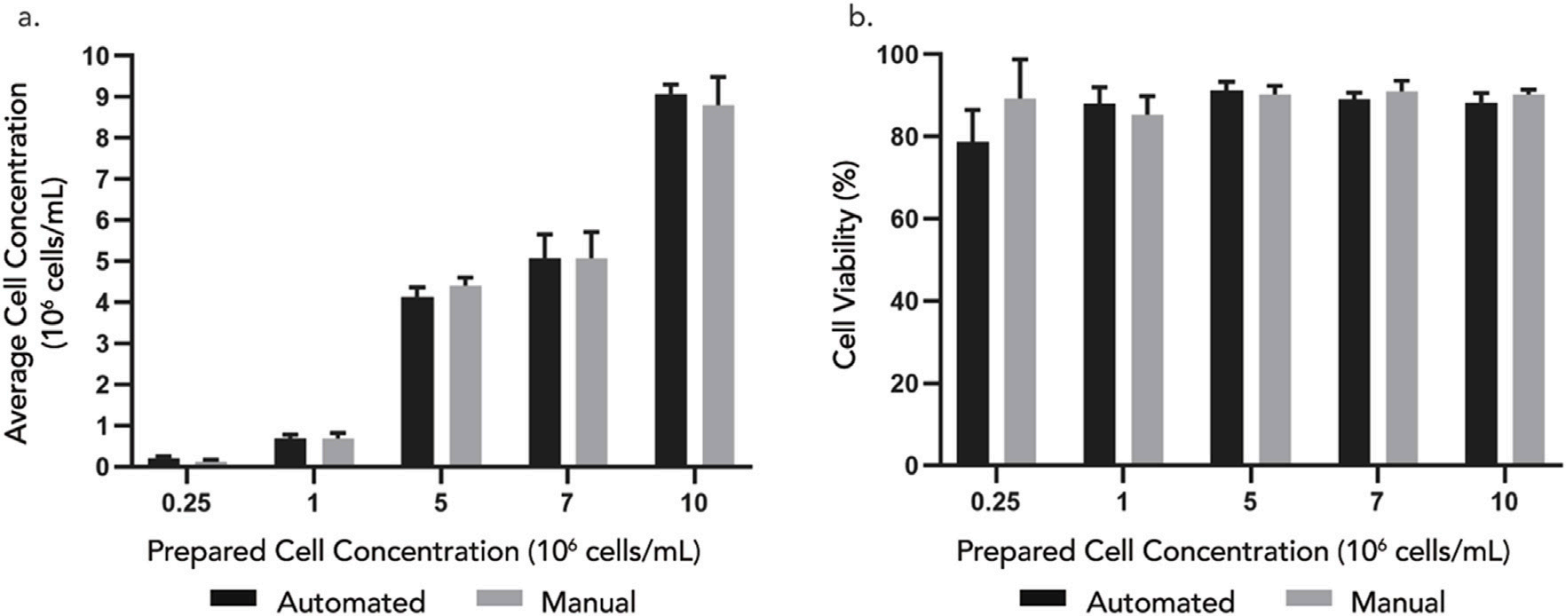
**(A)** Cell concentration and **(B)** viability of samples extracted from 5 different cell concentrations – 0.25, 1, 5, 7, 10 × 10^6^ cells/mL using automated DAAS versus manual pipetting. n = 3. Error bar stands for standard deviation. Student’s t-test indicates non-significant difference (P > 0.05) between the automated and manual group.

The results (Figure 6A) showed no statistically significant differences in cell concentration between samples extracted using DAAS and those obtained via manual pipetting for all the tested cell solutions. However, the cell concentrations of the extracted samples were lower than that of the stock cell solution by approximately 10%–30%, which was possibly due to insufficient homogenization prior to sampling or human error in the preparation of stock cell solutions. The standard deviation of cell concentration of samples extracted through DAAS was similar to or lower than that through manual sampling, indicating good consistency of the DAAS sampling compared to manual sampling. The small deviation also suggested sample carryover in the DAAS tubing was minimal and flushing or purging of the tubing pre-/post-sampling was unnecessary. Cell viabilities (Figure 6B) of the samples extracted from both sampling methods were similar and above 85%. This indicated that the DAAS sampling process did not negatively affect the cell viability. Overall, this study showcased the ability of the DAAS to sample comparably with the manual sampling method, achieving similar cell concentration and viability.

Manual sampling is labor intensive and exposes the culture to potential contamination risks. It is also concerned with potential process inconsistency which could compromise the quality of the sample analysis and subsequent process control strategy. DAAS overcomes these limitations of manual sampling, providing comparable sampling accuracy (cell density and viability) and additional benefits of automation, process consistency, and over the bench operation.

### Sampling during prolonged cell culture

DAAS can be especially advantageous when repeated sampling is required outside of BSC, for example, for continuous cell monitoring during prolonged cell culture. Two studies were conducted to demonstrate this DAAS application using two different cell types and cell culture platforms.

In Study 1, Jurkat cells were statically cultured in an open culture vessel for a continuous 1o days and cells were periodically sampled via DAAS or manual pipetting throughout the culture period. Cell growth and viability of the culture with DAAS sampling were compared with that of the culture with manual sampling. Sampling with the DAAS was conducted more frequently to explore the feasibility of using DAAS to provide regular samples for closer monitoring and tracking of cell growth with minimal disruption to the cell culture.

**FIGURE 7:**
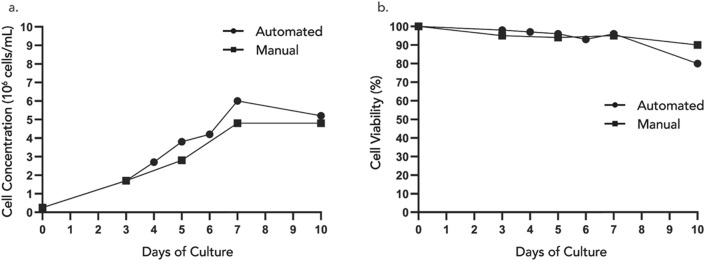
**(A)** Cell concentration and **(B)** viability of samples extracted automatically using DAAS versus manually using pipette in the BSC.

Results indicated that repeated sampling via DAAS did not compromise the sterility of the culture. Both culture groups achieved increasing cell growth, and similar and high cell viability of >90% from D0 to D7 (Figure 7). This suggested DAAS sampling did not negatively affect the cell growth when compared with manual sampling. After Day 7, the cell growth trend reversed in both groups to slow down or contact, and cell viability dropped. This indicated cell growth was nonideal after Day 7, which could be because the maximum cell density has been reached.

A closer look at cell growth revealed that Jurkat cells expanded from 0.25 × 10^6^ cells/mL on Day 0, to 6 × 10^6^ cells/mL with 24-fold increase in DAAS sampling group, versus 4.8 × 10^6^ cells/mL with 19.2-fold increase in the manual sampling group on Day 7 (Figure 7A). 20% more cell growth was obtained in DAAS sampling group compared to the manual group on Day 7 even when DAAS sampling was performed at a higher frequency. This difference in cell growth may be attributed to the reduced interruption to the culture caused by DAAS sampling, as the vessel remained inside the incubator under optimal culture conditions throughout the sampling process. The only interruption during this process was when the incubator door was opened for a short time (<20 s) to rock the culture vessel inside the incubator for homogenising the cell solution. In the manual sampling group, the interruption to the culture was longer (≥10 min) as the culture vessel had to be transferred between the incubator and the BSC. During the transfer and sampling, the culture was exposed to greater temperature, pH, and flow fluctuation that may have negatively impacted the cell growth.

Study 1 demonstrated that DAAS supported repeated in-process aseptic sampling without negatively affecting culture sterility or cell growth during a 10-day static Jurkat cell culture. It even led to higher cell yield by possibly reducing interruption to the cell culture compared to manual sampling.

In Study 2, donor T cells were expanded in an automated and closed bioreactor system (BECA-Auto) for 10 days and DAAS was used to automatically sample the cells during the culture. BECA-Auto is a standalone benchtop system with own environment control to gas, temperature and humidity akin to a conventional cell culture incubator. Due to the closed design, BECA-Auto can only be sampled with DAAS, but not manual sampling. For comparison, BECA-S (Open), the open and manual version of BECA-Auto, was also used to culture the same donor cells but in the incubator. Samples were extracted from BECA-S (open) manually in a Class II BSC. Cell growth and viability in BECA-Auto and BECA-S (Open) from two donor cell culture were compared.

**FIGURE 8:**
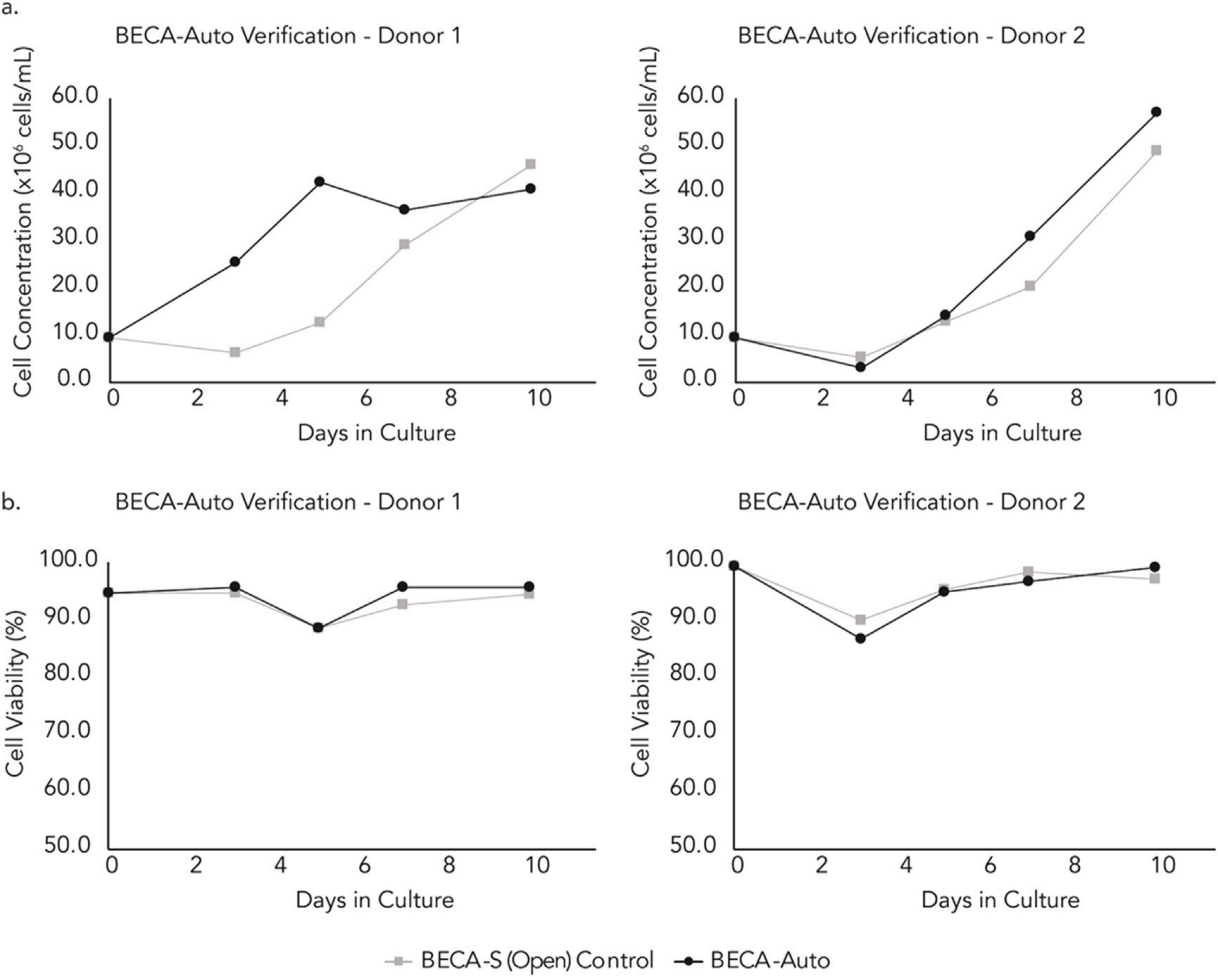
**(A)** Cell growth and, **(B)** Viability of samples extracted automatically from the BECA-Auto setup using DAAS, and manually from the BECA-S (Open) setup in the incubator (sampling conducted in the BSC).

Results indicated that repeated sampling via DAAS did not compromise the sterility of the culture in BECA-Auto for both donor cell cultures. Culture in both BECA-Auto and BECA-S (Open) exhibited increasing cell growth with BECA-Auto showing stronger growth compared to BECA-S (Open) for Donor 1 cell culture (Figure 8A). This showed that DAAS did not negatively impact the cell growth in BECA-Auto. The difference in cell growth in BECA-Auto and BECA-S (Open) might be attributed to the different sampling process and cell culture method. The unique sampling workflow and the close to zero dead volume of DAAS protected most of the culture in BECA-Auto from environmental changes (temperature/CO_2_/humidity), as only a small volume of culture was drawn out of the bioreactor and exposed to the environment outside of the incubation setup. In comparison, manual sampling to BECA-S (Open) transferred the culture vessel between an incubator and a BSC, and involved the removal of the vessel cap for sampling. Culture in BECA-Auto and BECA-S (Open) achieved similar and high cell viability >80% (Figure 8B), indicating the DAAS did not cause negative impacts to the cell viability.

Study 2 demonstrated DAAS’s suitability to be used for repeated in-process cell sampling from an automated and closed bioreactor for continuous donor T cell culture without negatively affecting the culture sterility, cell growth or cell viability.

These two case studies prove that DAAS can be used to sample multiple cell types from both open culture vessel and automated bioreactor system at multiple culture time points, supporting continuous cell culture monitoring during prolonged cell culture. It can sustain process sterility and high cell viability without causing adverse impacts to the culture.

## Future development

Further improvements can be made to the DAAS to make it a more complete solution for future cell and gene therapy manufacturing.

First, while we have successfully demonstrated the intended functionality of DAAS, the current form of the system is still a prototype and some of its hardware components have been chosen for quick prototyping and demonstration purpose rather than long term use. Moving forward, the DAAS’ components will be evaluated and replaced with more durable, reliable, compatible, and higher-grade alternatives for industrial purpose whenever desirable. For example, Arduino^®^ ATmega2560 microcontroller could be replaced with Programmable Logic Controllers (PLCs) for more reliable control of the automated sampling function.

Secondly, extracting a homogenised sample is important for providing an accurate representation of the cell culture population–which the DAAS is currently unable to do automatically. In the future, the DAAS can be integrated with an automated culture homogenization method such as having the culture vessel located on an automated platform within the incubation setup to rock, vibrate or swirl the culture.

In our experiments, an open microcentrifuge tube was used to collect the extracted samples at the outlet tubing of DAAS, which could not maintain sample sterility. However, maintaining the sterility of the extracted samples could be important for some applications such as sterility and contamination test. To facilitate this, the outlet tubing of DAAS can be connected with a closed sample manifold that contains multiple sample bags or syringes fitted with weldable tubing or aseptic connectors. This adaptation would necessitate outfitting the outlet tubing of DAAS with weldable tubing or aseptic connectors such as AseptiQuik (Colder Products Company, MN, United States), and the use of an additional fluid management module to direct the sample flow to a specific sample bag/syringe. After the samples are collected, the sample containing bag/syringe can be removed aseptically from the sample manifold by tube sealers or tubing disconnectors such as Clipster^®^ (Sartorius, Aubagne, France).

Interruptions to the cell culture process can be further minimised, which can be highly valuable for autologous cell and gene therapy manufacturing where the heterogenous cell source may be more sensitive to disruptions in culture. During DAAS operation, a small volume of culture is drawn out of the culture vessel/bioreactor and retained between the aseptic barrier of DAAS and the culture vessel/bioreactor. It is returned to the culture vessel only after the targeted sample volume is dispensed. While awaiting return, it is subjected to nonideal culture conditions (temperature/CO_2_/humidity) outside the incubator environment. Modifying DAAS’s control program to return this portion earlier and simultaneously with the dispensing of the targeted sample volume help reduce the exposure of the culture to suboptimal culture conditions. Another potential method involves adjusting the speed of the processes. These may entail reducing the pump speed during targeted sample volume formation to ensure volume accuracy, while increasing it for other processes like targeted sample volume extraction and returning culture to culture vessel. Whenever the manufacturing incubation system and process allow, DAAS can also be configured to be situated within the incubation system alongside the culture vessel, ensuring that the culture remains under optimal culture conditions at all times.

**FIGURE 9:**
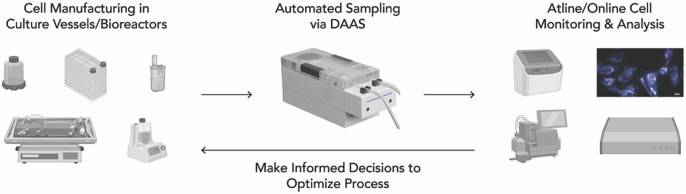
Integration of DAAS into a fully automated cell and gene therapy manufacturing workflow, where regular sampling for analysis potentially enables close cell culture monitoring and prompt in-process adaptive control.

In the long term, DAAS will be integrated with various cell monitoring and analysis equipment to provide manufacturers a comprehensive at-line/inline view of cell and culture conditions. For example, our collaborators from Singapore MIT Alliance for Research and Technology - Critical Analytics in Manufacturing Personalized-Medicine have demonstrated the integration of DAAS with a G-Rex vessel and a UV-Vis Spectrometer for real-time anomaly detection for Mesenchymal Stem Cell culture (Chelvam et al., 2022). Such integration will support the development of adaptive in-process controls, optimizing the cell and gene therapy manufacturing process and meeting product release criteria (Figure 9). This effort will accelerate the shift towards standardized, full connected and automated cell and gene therapy manufacturing. It will also help achieve timely delivery of qualified batches of cell and gene therapy products for urgent treatment protocols.

## Conclusion

DAAS has been developed to primarily enable autologous cell and gene therapy manufacturer for regular, automated, and aseptic small volume sampling of suspension cells or media. It has passed repeated bacterial ingression test and proved its aseptic sampling capability. Compared to common manual pipetting-based sampling, it has shown comparable sampling accuracy (cell density and viability) with minimal sample carry-over for sampling cell solutions of a wide range of cell concentrations. The application of DAAS for periodical sampling and continuous cell growth monitoring during prolonged cell culture has been successfully demonstrated with Jurkat cell culture in a static culture vessel, and donor T cell culture in a closed and automated bioreactor system. To summarize, DAAS carries great potential as a highly effective automated sampling solution for cell and gene therapy manufacturing. Its application facilitates close and automated cell monitoring and analysis, enabling informed and prompt decision-making to ensure the cells are developing as desired. This can potentially contribute to savings in costs, time, and labour in the cell and gene therapy manufacturing process.

## Data Availability

The original contributions presented in the study are included in the article/supplementary material, further inquiries can be directed to the corresponding author.
